# Investigation of the evolution of radiation-induced lung damage using serial CT imaging and pulmonary function tests

**DOI:** 10.1016/j.radonc.2020.03.026

**Published:** 2020-07

**Authors:** Catarina Veiga, Edward Chandy, Joseph Jacob, Natalie Yip, Adam Szmul, David Landau, Jamie R. McClelland

**Affiliations:** a Centre for Medical Image Computing, Department of Medical Physics & Biomedical Engineering, University College London, UK; b UCL Cancer Institute, University College London, UK; c Department of Respiratory Medicine, University College London, UK; d Department of Oncology, University College London Hospital, UK; e Department of Clinical Oncology, Guy’s & St Thomas’ NHS Foundation Trust, UK

**Keywords:** Lung, Radiation-induced lung damage (RILD), Computed tomography (CT), Pulmonary function test (PFT)

## Abstract

**Background and purpose:**

Radiation-induced lung damage (RILD) is a common consequence of lung cancer radiotherapy (RT) with unclear evolution over time. We quantify radiological RILD longitudinally and correlate it with dosimetry and respiratory morbidity.

**Materials and methods:**

CTs were available pre-RT and at 3, 6, 12 and 24-months post-RT for forty-five subjects enrolled in a phase 1/2 clinical trial of isotoxic, dose-escalated chemoradiotherapy for locally advanced non-small cell lung cancer. Fifteen CT-based measures of parenchymal, pleural and lung volume change, and anatomical distortions, were calculated. Respiratory morbidity was assessed with the Medical Research Council (MRC) dyspnoea score and spirometric pulmonary function tests (PFTs): FVC, FEV_1_, FEV_1_/FVC and DLCO.

**Results:**

FEV_1_, FEV_1_/FVC and MRC scores progressively declined post-RT; FVC decreased by 6-months before partially recovering. Radiologically, an early phase (3–6 months) of acute inflammation was characterised by reversible parenchymal change and non-progressive anatomical distortion. A phase of chronic scarring followed (6–24 months) with irreversible parenchymal change, progressive volume loss and anatomical distortion. Post-RT increase in contralateral lung volume was common. Normal lung volume shrinkage correlated longitudinally with mean lung dose (*r* = 0.30–0.40, *p* = 0.01–0.04). Radiological findings allowed separation of patients with predominant acute versus chronic RILD; subjects with predominantly chronic RILD had poorer pre-RT lung function.

**Conclusions:**

CT-based measures enable detailed quantification of the longitudinal evolution of RILD. The majority of patients developed progressive lung damage, even when the early phase was absent or mild. Pre-RT lung function and RT dosimetry may allow to identify subjects at increased risk of RILD.

## Highlights

• Detailed RILD evolution described by objective radiological and pulmonary function measures.
• RILD is associated with volume loss of the treated lung and contralateral lung hyperinflation.
• Objective radiological findings might differentiate subjects with early versus late RILD.
• Most patients developed progressive lung damage, even when the early phase is absent/mild.
• Pre-RT lung function and RT dosimetry may identify subjects at increased risk of developing RILD.

Radiation-induced lung damage (RILD) is a common complication of lung cancer radiotherapy (RT) [1]. RILD disrupts normal pulmonary physiology [2], reducing the quality of life of survivors [3], [4], [5], [6], [7]. Traditionally, RILD is separated into two phases: an acute phase (pneumonitis) during the first 6-months and a permanent phase (pulmonary fibrosis) >6-months post-RT [8], [9]. However, RILD is a dynamic process with acute and chronic inflammatory processes that are difficult to distinguish clinically; furthermore, it is unclear how the acute and chronic phases relate to each other [9], [10], [11], [12].

Radiological findings provide critical information on the post-RT evolution of the respiratory system that is complementary to functional and symptomatic information. Imaging endpoints in particular allow the definition of objective measures that facilitate quantification and clinical correlation [3], [13], [14], [15], [16], [17], [18]. Thus, computed tomography (CT) imaging is commonly used to study RILD [8], [19], [20]. Although impairment of pulmonary function is common in survivors [4], [7], correlating imaging findings and clinical symptoms has been challenging [3], [16], [17] likely due to various confounding factors that add complexity to the study of RILD (including pre-existing lung conditions and use of combination therapies) [21]. A more comprehensive evaluation of radiological findings is necessary to distinguish acute and chronic inflammation, informing our understanding of underlying pathophysiology that occurs in the lung post-RT which may facilitate the development of personalised therapeutic interventions. The long-term effects of RILD merit increased consideration as lung cancer treatment and survival improves [22], [23], [24], [25] and the use of immune checkpoint inhibitors in radically treated patients increases [26], [27].

The aim of this study is to quantify the longitudinal evolution of RILD during the first 24-months after RT and correlate it with dosimetry and respiratory morbidity. We use clinical pulmonary functions tests (PFTs) together with a suite of novel quantitative CT-based measures [15] to describe the evolution of RILD. We expect our objective and comprehensive analysis to enrich our current understanding of how RILD develops and evolves, and to provide new insights that inform future, prospective studies of RILD.

## Methods and materials

### Study group

Data from subjects treated in a multicentre, non-randomized, phase 1/2 chemoradiation trial of stage II/III non-small cell lung cancer (IDEAL-CRT) were included in this study [24]. RT was planned isotoxically (mean lung dose of 18.2 Gy in equivalent dose in 2 Gy fractions) with tumour doses escalated up to 73 Gy. RT was delivered in 30 fractions over 6 weeks (5 fractions per week) or 5 weeks (6 fractions per week, with one day a week of two fractions), with two cycles of concurrent cisplatin and vinorelbine. Most RT plans were 3D conformal (98%).

### CT scans and pulmonary function tests

Protocol called for CT scans and PFTs to be performed pre-RT and at fixed time-points post-RT (3, 6, 12 and 24-months) in all patients. Of the 120 patients treated in IDEAL-CRT, 51 had CT scans at all timepoints collected centrally. We excluded patients due to poor CT quality (4), complete lung collapse (1), and with missing dosimetry (1), leaving 45 datasets for analysis (Table 1).

**Table 1:** Patient characteristics.

Sex, no. (%)						
	Male	30 (67%)				
	Female	15 (33%)				

Age (y), median (range)						
	64 (42–83)					

Staging, no. (%)						
	IIB	3 (7%)				
	IIIA	29 (64%)				
	IIIB	13 (29%)				

Radiotherapy dosimetry details (Gy), median (range)						
	Prescription dose	67.5 (63.0–73.0)				
	Mean lung dose	14.5 (8.8–20.0)				
	Mean heart dose	9.9 (1.1–30.8)				

PTV size (cm^3^), median (range)						
	365 (139–821)					

Fractionation scheme, no. (%)						
	6-week protocol	35 (78%)				
	5-week protocol	10 (22%)				

Radiotherapy technique, no. (%)						
	Conformal	44 (98%)				
	IMRT/VMAT	1 (2%)				

Recurrence status at 24-months, no. (%)						
	Any location	12 (27%)				
	Locoregional					
	within RT volume	9 (20%)				
	outside RT volume	3 (7%)				
	Distant	5 (11%)				

CT imaging resolution (mm), median (range)						
	0.79 × 0.79 × 2.00 (0.57 × 0.57 × 0.50–1.37 × 1.37 × 5.0)					

Time to imaging session since RT end (days), mean ± std						
	3-months	80 ± 7				
	6-months	172 ± 15				
	12-months	340 ± 25				
	24-months	707 ± 47				

Clinical tests available, no. (%)						

		MRC^1^	FVC^2^	FEV_1_^3^	FEV_1_/FVC^4^	DLCO^5^

	Pre-RT	43 (96%)	45 (100%)	44 (98%)	44 (98%)	45 (100%)
	3-months	42 (93%)	41 (91%)	41 (91%)	41 (91%)	37 (82%)
	6-months	44 (98%)	38 (84%)	38 (84%)	38 (84%)	36 (80%)
	12-months	43 (96%)	38 (84%)	38 (84%)	38 (84%)	38 (84%)
	24-months	42 (93%)	34 (76%)	34 (76%)	33 (73%)	33 (73%)

Pre-RT MRC dyspnoea score, median (range)						
	1 (0–3)					

Pre-RT pulmonary function, median (range)						
	FVC (%pred)	95 (70–132)				
	FEV_1_ (%pred)	77 (37–117)				
	FEV_1_/FVC (%)	67 (36–87)				
	DLCO (%pred)	67 (42–111)				

^1^MRC = Medical Research Council dyspnoea score.

^2^FVC = Forced vital capacity.

^3^FEV_1_ = Forced expiratory volume in 1 s.

^4^FEV_1_/FVC = Tiffeneau-Pinelli Index.

^5^DLCO = Diffusion capacity for carbon monoxide (CO).

Respiratory morbidity was routinely assessed with spirometric PFTs and Medical Research Council (MRC) dyspnoea scores (Table 1). MRC qualitatively grades how breathlessness affects day-to-day activities in a five-point scale [28] while PFTs quantitatively measure pulmonary function. A data cleansing protocol was applied to the PFT data (supplementary material A). PFT change at follow-up (F) was expressed as relative difference from pre-RT (baseline, B) measured values, i.e., $\Delta PFT=100\times \left(PFT_{F}-PFT_{B}\right)/PFT_{B}$; MRC is expressed as absolute difference ($\Delta MRC=MRC_{F}-MRC_{B}$). PFT toxicity was graded according to Radiation Therapy Oncology Group (RTOG) [29].

### Radiological features of RILD

We recently developed a suite of twelve semi-automated, quantitative CT-based biomarkers of RILD to measure common post-RT radiological findings (parenchymal, pleural and lung volume changes) [15], [20]. The biomarkers provide a detailed, continuous description of RILD well beyond commonly used local density changes in the lung parenchyma [14], [18], [30], [31], [32], [33], [34], [35]. Table 2 summarises the calculated measures; details on implementation, evaluation and limitations have been previously described [15]. Briefly, CT images acquired pre- and post-RT are rigidly aligned. Regions of anatomical interest are first automatically segmented and then manually revised by a radiation oncologist and/or physicist (EC/CV). Objective anatomical features are measured at each time-point from the CT images and segmentations. Some features (NV, RV, X, Z, C, α and M) are normalised by the corresponding feature measure in the contralateral lung to account for variation in inhalation level between scans and (except for RV) converted to a percentage. The biomarkers (except for RV) are then defined as the absolute or relative change in the features at follow-up from pre-RT value. These biomarkers measure actual radiological change and are not surrogates of other endpoints. To complement analysis on post-RT volume loss, we also calculated the relative change (from pre-RT value) of the normal contralateral, ipsilateral and total lung volumes (ΔCV, ΔIV and ΔTV, Table 2).

**Table 2:** Summary of the CT-based imaging measures.

Symbol	Name	Description [units]
ΔNV	Normal lung volume shrinkage	Reduction in normal lung volume, defined from total lung volume by applying a threshold of intensities (HU ≤ −500)* [%]
RV	Volume of consolidation	Ratio of high-intensity volume versus total lung volume at follow-up (i.e., measure of parenchymal change, mostly consolidation)* [1]^+^
ΔX	Lung width reduction	Reduction in maximum lung width* [%]
ΔZ^§^	Lung height reduction	Reduction in maximum lung height* [%]
Δh	Diaphragmatic elevation	Change in the height difference between ipsilateral and contralateral diaphragms [mm]
ΔC	Diaphragmatic curvature	Change in the curvature of the diaphragm surface* [%]
ΔS	Diaphragmatic tenting	Increase in the surface area of diaphragm tented [mm^2^]
Δα	Main bronchus rotation	Main bronchus rotation in coronal view* [%]
ΔM	Mediastinal shift	Shift of the carina toward the ipsilateral lung* [%]
Δβ	Anterior junction line rotation	Anterior junction line rotation toward the ipsilateral lung [°]
Δt	Anterior junction line thickening	Ratio between the thickness of the anterior junction line at follow-up and baseline [1]^+^
ΔP	Pleural change	Increase in the surface of the chest wall covered with pleural reactions [%]
ΔCV	Normal contralateral lung volume shrinkage	Relative change (from pre-RT value) of the normal contralateral volume^$^ [%]
ΔIV	Normal ipsilateral lung volume shrinkage	Relative change (from pre-RT value) of the normal ipsilateral lung volume^$^ [%]
ΔTV	Normal total lung volume shrinkage	Relative change (from pre-RT value) of the normal total lung volume^$^ [%]

*Relative to contralateral lung. For example, the biomarker “normal lung volume shrinkage” (ΔNV) is defined as the difference between the value measured for the anatomical feature “normal lung volume” at baseline and at follow-up (${NV}_{B}-{NV}_{F}$, with B = baseline, F = follow-up). NV is normalized by the equivalent measure from the contralateral lung and converted to a percentage. Therefore, $\Delta NV=100\times \left({NV}_{B,i}/{NV}_{B,c}-{NV}_{F,i}/{NV}_{F,c}\right)$_._

^+^[1] = dimensionless.

^§^Definition was updated from the original publication [15].

^$^For example, “Normal contralateral lung volume shrinkage” is defined as $\Delta CV\left[\%\right]=100\times \left({NV}_{B,c}-{NV}_{F,c}\right)/{NV}_{B,c}$. Absolute lung volume varies considerably with inhalation level (exhale vs inhale scans), so these measures are not robust when baseline and follow-up scans are acquired with inconsistent inspiration.

### Analysis

All radiological measures were calculated at serial time-points for all subjects. The time-dependent relationships of the radiological findings, RT dosimetry and PFTs were then investigated in detail. Statistical analysis was performed using MATLAB 2019a Statistical Toolbox. Due to the exploratory nature of this study, the statistical significance level was set as 10%. Corrections for multiple comparison adjustment were done using Benjamini-Hochberg procedure (10% false discovery rate). Since not every patient had complete datasets (i.e., all radiological measures and PFTs at all time-points), the dimensions of the samples used in different analyses were variable.

## Results

**Fig. 1:**
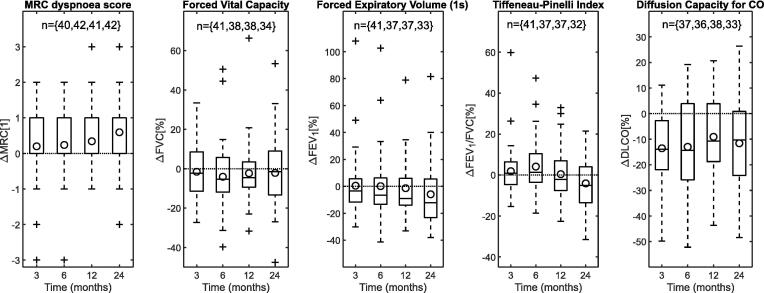
Post-RT change in MRC score and PFTs. Circles indicate average value; horizontal line indicates no change; outliers fall outside the ±2.7 std range.

The time-dependent changes in MRC dyspnoea score and PFTs are shown in Fig. 1. The incidence of grade 1+ PFT toxicity calculated according to RTOG (i.e., declines >10% in PFTs) was 32%, 55% and 48% at 24-months for FVC, FEV_1_ and DLCO, respectively; no grade 3 events or higher (i.e., declines >50% in PFTs) were calculated. The MRC score progressively worsened over time. FVC decreased at earlier time-points but from 12-months recovered partially to pre-RT values. FEV_1_ and FEV_1_/FVC were unchanged on average at 3-months from pre-RT values, and then decreased progressively. The decline in DLCO from pre-RT was significant at all time-points (Wilcoxon paired two-sided signed rank tests with multiple comparison adjustment, *p* < 0.01) but was not progressive. Changes in MRC score at 12 and 24-months (from baseline readings) were statistically significant (*p* = {0.03,0.01}); FVC changes were significant at 6 and 12-months (*p* = {0.04,0.03}); FEV_1_ changes were significant at 24-months (*p* = 0.02). MRC changes, which are related to symptoms and patient well-being, were linked mostly with decline in volume-based spirometry (Pearson’s correlation coefficient *r* = {-0.41, -0.45}, *p* = {<0.01, <0.01} for FVC and FEV_1_, respectively). Complete data shown in supplementary material B (Tables S.1 and S.2, and Fig. S.1).

**Fig. 2:**
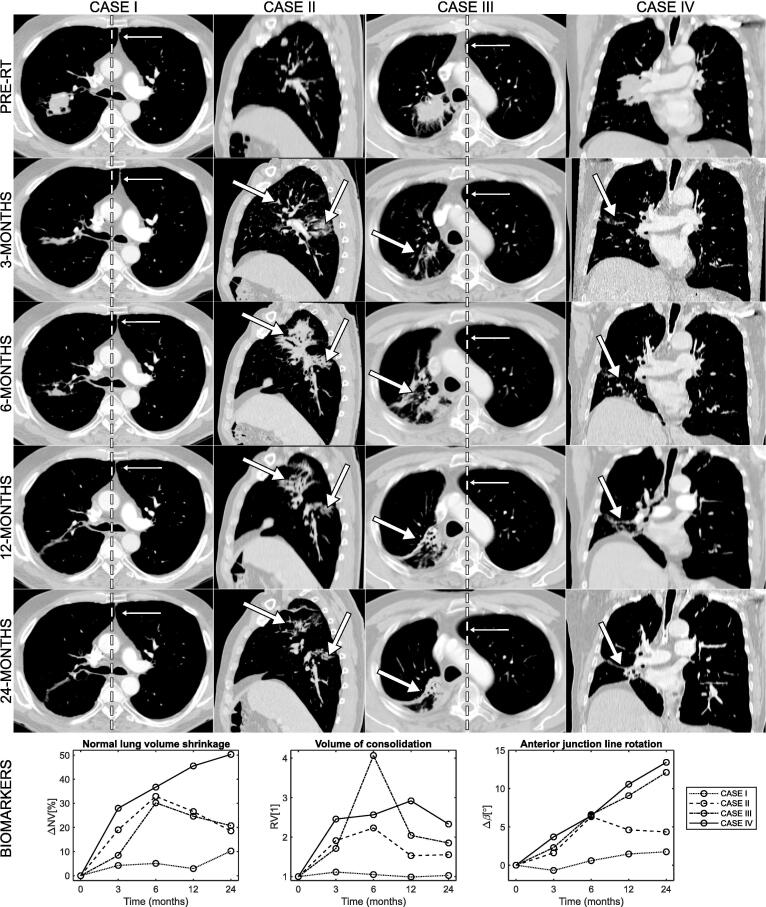
Examples of evolution (from pre-RT to 24-months post-RT) of radiological changes and corresponding values calculated for selected CT-based biomarkers. Case I: The observed changes, which included parenchymal damage, lung volume shrinkage and anatomical distortions, were mild but measurable, and worsened over time. The dashed line highlights the evolution of rotation of the anterior junction line (Δβ) indicative of volume loss with anatomical distortions. Case II: Diffuse parenchymal change was visible at 3-months (arrow); at 6-months those regions evolved into dense consolidation. Most parenchymal changes (RV) visually resolved by 24-months. Anatomical distortions were modest and did not worsen after 6-months (Δβ). The pronounced loss in normal lung volume (ΔNV) at 6-months was explained primarily by inflammatory changes and was not permanent. CASE III: Normal lung volume loss and consolidation volume peaked at 6-months (ΔNV and RV). As time progressed, the regions of consolidation shrank and became denser while anatomical distortions were increasingly evident (Δβ). The observations reflecting inflammation tended to resolve whilst those reflecting scarring progressed. CASE IV: Lung volume loss progressively worsened with time (ΔNV), with anatomical distortions also becoming more apparent as time passed (Δβ). This is compatible with a dominant effect of permanent fibrotic scarring. The increase in high-intensity lung volume remained stable across time-points although there was progressive lung volume shrinkage (RV and ΔNV). This was due to the presence of a residual mass that shrank across follow-up time-points in parallel with the scarring process. Complementary projections in supplementary material B (Fig. S.2).

**Fig. 3:**
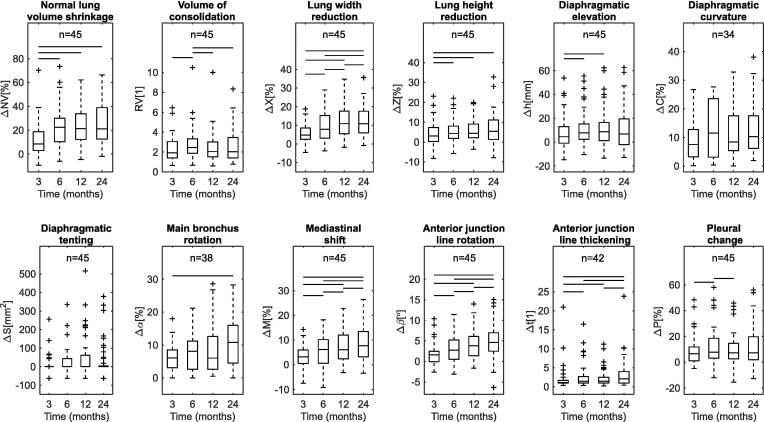
Post-RT CT-based biomarkers. Cases of non-measurable Δt, Δα and ΔC were due to toxicities at the junction line, blocked airways and artefacts in the diaphragm. Horizontal lines indicate statistically significant differences (pairwise Wilcoxon two-sided signed-rank tests after Benjamini-Hochberg procedure, 10% false discovery rate); outliers fall outside the ±2.7 std range.

Radiological findings of RILD appeared and evolved during the 24-months after RT. Fig. 2 shows some illustrative cases. The range of values measured per biomarker at serial time-points is shown in Fig. 3. Radiological change was present from 3-months. Parenchymal change (measured by RV) was common at 3-months and peaked at 6-months, then reduced from 6 to 24-months. On visual inspection, parenchymal changes evolved from ground-glass opacities at 3-months to denser consolidation patterns, consistent with the development of scarring (e.g. case III, Fig. 2). The affected lung was seen to partially collapse from 6-months onwards (20% incidence at 24-months), possibly due to airway stenosis, fibrotic retraction or local recurrence (20% in-field recurrence at 24-months, Table 1). Normal lung volume shrinkage (ΔNV) and most measures of anatomical distortion (ΔX, ΔZ,Δα, ΔM, Δβ and Δt) became more severe over time, peaking at 24-months. The remaining measures of anatomical distortion peaked earlier and stabilised, with Δh and ΔS stabilising between 12 and 24-months, and ΔC recovering after 6-months. Pleural change (ΔP) was common at all time-points but its evolution varied across the patient group.

Friedman test identified significant changes between timepoints for 10 out of the 12 biomarkers (*p* ≤ 0.10). Post-hoc Wilcoxon two-sided signed-rank tests with multiple comparison adjustment were used to identify significant changes between time-points. The most pronounced variations occurred from 3 to 6-months, where 9 out of 12 biomarkers showed statistically significant changes. Changes in ΔX, ΔM and Δβ were statistically significant between all time-points, while changes in ΔS and ΔC did not reach significance between any time-points. All p-values reported in supplementary material B (Table S.3).

Longitudinal worsening of ΔIV and ΔTV indicate loss of ipsilateral and total lung volume. Results for ΔCV suggest a systematic increase in volume of the contralateral lung post-RT. At 24-months, the contralateral volume increased in 67% of the subjects. However, the change from pre-RT values did not reach statistically significant levels (Wilcoxon two-sided signed-rank test with multiple comparison adjustment, *p* = {0.19, 0.46, 0.10, 0.11} for *t* = {3, 6, 12, 24}-months). Full data is shown in supplementary material B (Fig. S.3 and Table S.4).

**Fig. 4:**
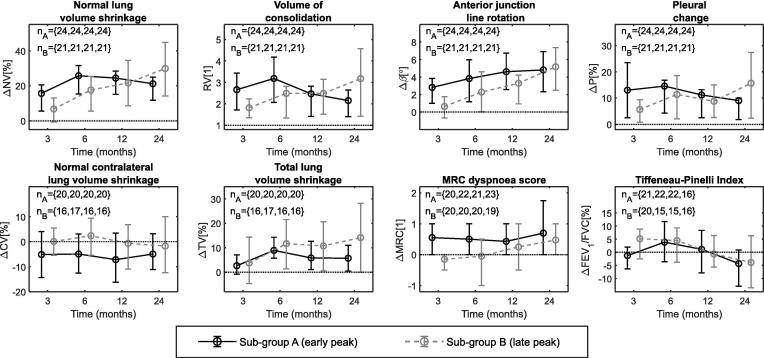
Average value (±25/75% percentile) for selected radiological and PFT data, per sub-group at serial time-points; horizontal line indicates no change. Full data shown in supplementary material B (Fig. S.3).

Fibrotic damage associated with chronic inflammation often results in permanent lung shrinkage whereas acute inflammation disappears with time and normal lung volume partially returns to previous values. To investigate whether the radiological findings could distinguish acute from chronic changes, we divided the patient group into two sub-groups according to the evolution of ΔNV. Sub-group A (early peak) included 24 subjects where ΔNV was most severe at 3–12-months. Sub-group B (late peak) included the remaining 21 subjects whose ΔNV was most severe at 24-months. We then compared radiological and PFT data for these two sub-groups (Fig. 4, supplementary material B Fig. S.4).

On average, patients in sub-group A exhibited larger values for the biomarkers up to 6-months: ΔNV and RV peaked at 6-months and then became less severe; ΔP was common at earlier time-points but tended to resolve over time; the remaining biomarkers, which predominantly reflected lung volume loss with anatomical distortions, stabilised or recovered between 6 and 24-months; recovery in ipsilateral lung volume (ΔIV) and increased contralateral lung (ΔCV) volume lead to less severe long-term total volume loss (ΔTV). MRC scores worsened earlier after RT (and then stayed constant). In sub-group B all biomarkers (except for ΔC and ΔS) and MRC scores became progressively more severe over time; in general, sub-group B reached by 24-months similar (or higher) values to sub-group A.

We found evidence of differences in pre-RT values for MRC scores, FVC and DLCO (percent predicted values) between the sub-groups (Wilcoxon two-sided rank-sum test, *p* = {0.01, 0.06, 0.01}), with sub-group B having in general poorer PFTs pre-RT. We found no other significant differences between the two groups when tested for other pre-RT factors (including age, prescription, lung and heart dose metrics, GTV size, FEV_1_ and FEV_1_/FVC). Data shown in supplementary material B (Table S.5).

The relationship between the radiological biomarkers and RT dosimetry was investigated. Lung volume shrinkage (ΔNV and ΔIV) over time correlated consistently and most strongly with global RT dosimetry; correlations were generally moderate although statistically significant. For example, ΔNV correlation with MLD ranged between *r* = 0.30–0.40 (Pearson’s correlation coefficient, *p* = 0.01–0.04) over all time-points. Correlations with dosimetry are likely obscured by the isotoxic RT design. Data shown in supplementary material B (Fig. S.5).

We also investigated the relationship between the time-dependent radiological findings and respiratory morbidity. Data from all subjects at all time-points was pooled for analysis. FVC and FEV_1_ changes correlated consistently but modestly with radiological measures of lung volume loss (ΔNV, ΔIV and ΔTV). For example, ΔFVC correlations of *r* = -0.22 were found for ΔNV (*p* = 0.01), *r* = -0.43 for ΔTV (*p* < 0.01), and *r* = -0.14 for Δβ (*p* = 0.08). Lung volume loss correlated better with FVC and FEV_1_ when it was not normalised to the contralateral side (ΔIV and ΔTV) than when it was (ΔNV). ΔFEV_1_/FVC had poorer correlation with volume changes and correlated best with mediastinal rotations: *r* = -0.12 for ΔNV (*p* = 0.17), *r* = -0.04 for ΔTV (*p* = 0.67), and *r* = -0.29 for Δβ (*p* < 0.01). DLCO generally correlated poorly with radiological findings.

It is likely that correlations with PFTs are obscured by heterogeneous sub-groups. We noticed that biomarkers in sub-group B correlated more strongly with PFTs than sub-group A. For example, a correlation of *r* = -0.43 was found between ΔFVC and ΔTV (*p* < 0.01) when considering all subjects; the correlation was *r* = 0.03 for sub-group A subjects only (*p* = 0.78), and *r* = -0.67 for sub-group B (*p* < 0.01). This disparity between sub-groups was consistently found for other biomarkers and PFTs. Data shown in supplementary material B (Fig. S.6). These findings hence suggest differing radiological evolution patterns post-RT with differing functional patterns in the radiologically-stratified sub-groups.

## Discussion

In this study we demonstrate the use of CT-based imaging biomarkers, together with PFTs, to investigate the evolution of RILD in patients treated with isotoxically dose-escalated 3D-CRT. To the best of our knowledge, this is the first time RILD up to 24-months post-RT has been described in such detail in radically treated patients. We have demonstrated that a variety of intuitive semi-automated radiological measures of parenchymal, lung volume and pleural change can be used to characterise reversible and long-term lung damage which are not quantifiable by human observers. Hyperinflation of the contralateral lung is identified as a potential consequence of RILD. The ability of the biomarkers to capture fine details of RILD morphology and of distinguishing differing longitudinal patterns of lung damage is confirmed.

Our findings indicate an evolution of RILD from predominantly acute inflammation, characterised by early (3–6 months) reversible parenchymal change (RV) and non-progressive anatomical distortion, into chronic inflammatory scarring (6–24 months), characterised by irreversible parenchymal change, progressive lung volume loss (ΔNV) and anatomical distortions (ΔX, ΔZ, Δα, ΔM, Δβ and Δt). Our findings are consistent with the study by Bernchou et al. (2013) investigating parenchymal change in 131 NSCLC patients receiving IMRT, where they describe a dose-dependent evolution consistent with the superposition of early (pneumonitis) and late (fibrosis) components, mathematically modelled using skewed bell and sigmoid shape functions [34].

The evolution of ΔNV guided the separation of the study population into two sub-groups based purely on radiological findings. The sub-grouping differentiated subjects with predominantly acute inflammatory reactions versus patients with mostly persistent fibrotic RILD. Our study provides quantitative evidence that the majority of subjects progressed to develop late RILD, even when imaging findings were absent or mild in the early phase [9], [11]. Patients in the late change group had poorer pulmonary function pre-RT. We believe our suite of biomarkers to be a valuable tool to test hypotheses and guide future investigations into the loss of lung function post-RT [36]. For example, Kong and Wang discuss how patients with poorer spirometry may tolerate RT better than patients with normal function [21], [37]. They speculate that COPD may protect against radiation toxicity as emphysematous lung contains less parenchymal tissue and has poorer cellular oxygenation.

Lung volumes change as consequence of RT. Our data indicates a trend toward contralateral lung expansion after RT. This effect may have been overlooked historically by a focus on post-RT total lung volume loss. Further investigation on its clinical impact is warranted as hyperexpansion of non-irradiated regions may not necessarily improve gas exchange and/or lung mechanics [11], [38].

Decline in pulmonary function after RT is common and time-dependent. Most subjects report long-term impairment of pulmonary function. Lopez Guerra et al. describe similar temporal patterns for FEV_1_/FVC and DLCO, reporting average declines of 3.7% and 17%, respectively, at 9–12 months [4]. Torre-Bouscoulet et al. report serial lung function up to 48 weeks after 3D-CRT, and also found a significant reduction in total lung capacity and PFT deterioration [7]. We found that FVC partially recovers, which might relate to inflammation abating after 6-months. Decline in FVC and FEV_1_ correlated with change in MRC scores and radiological lung volume loss. The progressive decline in FEV_1_ and FEV_1_/FVC suggests long-term obstructive airways disease that did not correlate with lung volume loss but which linked to biomarkers reflecting progressive mediastinal distortion. Although we found modest correlations between radiological findings, dosimetry and PFTs, similar to other studies [16], there is evidence that differing functional trends between population sub-groups obscures these relationships.

Our study has certain limitations. The number of patients in our analyses allows demonstration of quantitative trends but precludes the development of firm conclusions. We only included patients that survived 24-months as we wanted to study the longitudinal evolution of RILD. This inclusion criterion is likely to have excluded cases where severe radiological and respiratory changes occurred earlier and may have affected morbidity and therefore patient follow-up. PFTs and MRC scores only allow crude characterisation of a patient’s functional and symptomatic status. Likewise, whilst the biomarkers describe a wide spectrum of radiological change, they only provide measures of damage at a global scale. Further work is necessary to comprehensively describe damage at a regional level. We have also not distinguished parenchymal features such as consolidation, ground-glass opacities, reticulation and traction bronchiectasis [39]. When RILD evolves, these patterns can develop from one type to another. The extent of damage may remain constant despite its pathophysiological phenotype altering. More nuanced classification of parenchymal features should enhance our understanding of the morphological evolution of lung damage post-RT. A degree of uncertainty is also attributable to CT segmentation errors and variability in inhalation level, scan quality and acquisition [15]. Future work should address these current limitations by investigating larger patient cohorts, expanding the suite of biomarkers to measure different types of parenchymal change [14], [32], [33] and fully automating the required pipelines. Prospective studies are needed to allow inhalation levels and image acquisition to be standardised and should include comprehensive patient reported measures of respiratory symptoms and function.

In summary, we have quantified the evolution of radiological RILD and shown how it relates to RT dosimetry and respiratory morbidity. The key findings of our study are: (1) detailed radiological measures allow tracking and separation of acute and chronic patterns of RILD; (2) RILD is associated with hyperexpansion of the contralateral lung, which may be clinically relevant; (3) the majority of lung cancer survivors develop progressive RILD, even when early phase damage appears absent or mild; (4) pre-RT PFTs may help identify sub-groups at risk of early acute RILD; (5) global radiological damage is linked with higher mean lung RT doses; (6) post-RT radiological lung volume loss is linked with decline in volume-based spirometry. These findings should be tested prospectively in larger cohorts.

## Conflict of interest statement

CV reports other support from charitable donation, during the conduct of the study. EC reports other support from charitable donation, during the conduct of the study. JJ reports personal fees from Roche, personal fees from Boehringer Ingelheim, outside the submitted work. AS reports other support from charitable donation, during the conduct of the study. JRM reports other support from charitable donation during the conduct of the study, and support from Elekta outside the submitted work.
