# Supplementary material for: Investigation of the evolution of radiation-induced lung damage using serial CT imaging and pulmonary function tests
Source: Radiother Oncol. 2020 Jul;148:89–96. doi: 10.1016/j.radonc.2020.03.026 (PMC7416106; doi:10.1016/j.radonc.2020.03.026)
Supplement: Supplementary data 1 [file mmc1.docx]

SUPPLEMENTARY MATERIAL A

**Methods & Materials – Pulmonary function test (PFT) data cleansing**

PFT data collected centrally included the measured value, predicted value, and percent predicted value for FVC, FEV_1_ and DLCO. FEV_1_/FVC (also called Tiffeneau-Pinelli Index) is derived from FVC and FEV_1_. These three recorded measures are linked by the mathematical expression:

$$\mathrm{PFT}_{\%predicted}\left[ \% \right]=100\times\frac{\mathrm{PFT}_{\mathrm{measured}}}{\mathrm{PFT}_{\mathrm{predicted}}}$$

Upon initial QA of the PFTs database, we identified inconsistencies and implausibility in the raw PFT data where:

- the mathematical formula above was violated;
- FEV_1_ values were higher than FVC;
- DLCO predicted values were biologically implausible, hinting the use of different set of units (traditional mL/min/mm Hg vs SI mmol/min/kPa)

These all indicated insertion errors in at least one of these three measures. Therefore, a data cleansing protocol was applied to deal with such issues. The data was queried to identify cases with:

- errors larger than 5% in $\mathrm{PFT}_{\%predicted}$, a threshold that could be explained by the precision used in the reporting (i.e., due to rounding numbers);
- FEV_1_>FVC;
- predicted DLCO<10, indicating SI instead of traditional units.

First, we attempted to correct errors by re-requesting data from the local cancer centres via the central clinical trials unit. Then, any outstanding discrepancies were excluded from analysis unless one of the two records of predicted PFT (predicted value and percent predicted value) could be discarded individually due to biological implausibility. In every case, EC and CV independently reviewed the data and reached consensus if queried data points should be included or excluded.

Out of the 900 measured values of the 4 different PFTs (FVC, FEV_1_, FEV_1_/FVC and DLCO) for all 45 subjects at 5 timepoints (pre-RT and 3, 6 12 and 24-months after RT), 101 were not collected centrally and 25 were excluded after data cleansing, leaving a total of 774 for analysis (of which 2 were queried but included).

SUPPLEMENTARY MATERIAL B

**Results**

Table S.1- P-values for Wilcoxon tests with multiple comparison adjustment (using Benjamini-Hochberg procedure, 10% false-discovery rate) for MRC score and PFTs with respect to pre-RT values. Values in bold indicate statistically significant results.

| Pulmonary function test | Pairwise Wilcoxon two-sided signed rank-test | | | | |  |
| --- | --- | --- | --- | --- | --- | --- |
|  | critical  p-value | uncorrected p-value | | | |  |
|  |  | 0-3M | 0-6M | 0-12M | 0-24M | |
| MRC | 0.03 | 0.20 | 0.11 | **0.03** | **0.01** | |
| FVC | 0.04 | 0.24 | **0.04** | **0.03** | 0.25 | |
| FEV_1_ | 0.02 | 0.12 | 0.25 | 0.06 | **0.02** | |
| FEV_1_/FVC | 0 | 0.82 | 0.15 | 0.76 | 0.06 | |
| DLCO | <0.01 | **<0.01** | **<0.01** | **<0.01** | **<0.01** | |

Table S.2- Decline in pulmonary function calculated according to RTOG PFT Toxicity Scale.

|  |  | Incidence at follow-up | | | |
| --- | --- | --- | --- | --- | --- |
| Pulmonary Function Test | RTOG grade^*^ | 3M | 6M | 12M | 24M |
| FVC | Grade 0  Grade 1  Grade 2  Grade 3+ | 71%  27%  2%  0% | 68%  18%  13%  0% | 76%  21%  3%  0% | 68%  26%  6%  0% |
| FEV_1_ | Grade 0  Grade 1  Grade 2  Grade 3+ | 71%  27%  2%  0% | 54%  38%  8%  0% | 54%  41%  5%  0% | 45%  36%  18%  0% |
| DLCO | Grade 0  Grade 1  Grade 2  Grade 3+ | 41%  35%  24%  0% | 47%  28%  25%  0% | 47%  37%  16%  0% | 52%  24%  24%  0% |
| *grade 1: 10%–25% decline; grade 2: >25%–50% decline; grade 3: >50%–75% decline; grade 4: >75% decline; grade 5: death). | | | | | |

**
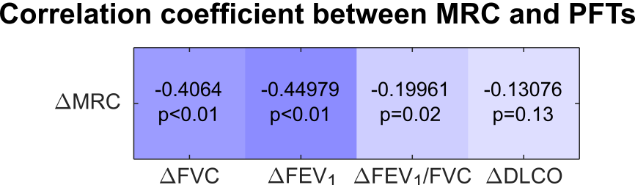
**

Figure S.1- Pearson’s correlation coefficient between MRC and PFTs. Data from all subjects at all time-points was pooled for analysis.


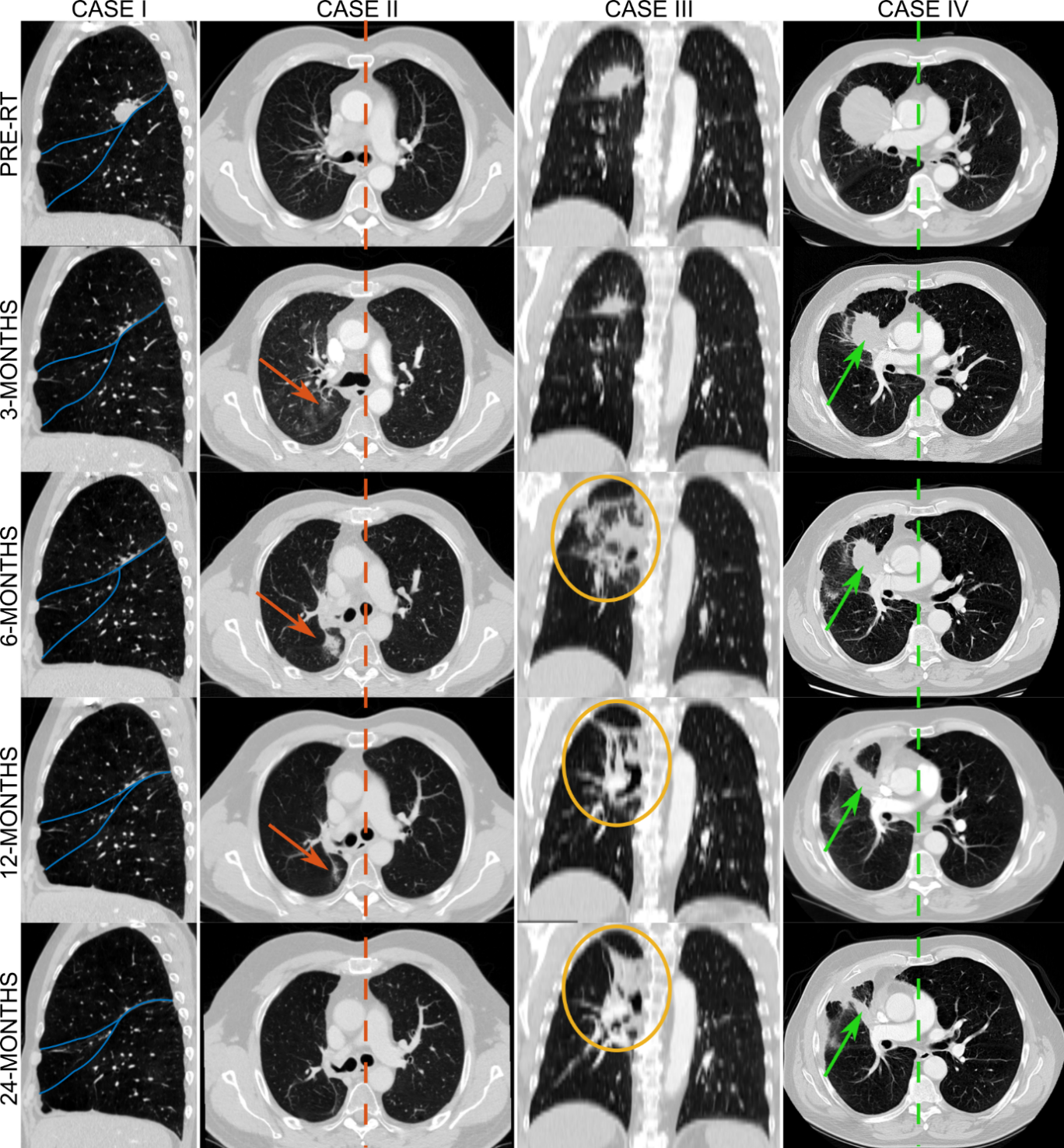


Figure S.2- Examples of evolution (from pre-RT to 24-months post-RT) of radiological changes in four subjects – complementary projections to Figure 1 (i.e., same subjects on different imaging views). Case I: While lung volume reduction was mild, we can identify changes in the relative volume of the different compartments of the lung changes by 24-months (fissures identified). Case II: Diffuse parenchymal change was visible at 3-months (arrow); at 6-months those regions evolved into dense consolidation. Most parenchymal changes visually resolved by 24-months. Anatomical distortions were modest and did not worsen after 6-months (dashed line highlights the evolution of rotation of the anterior junction line). CASE III: Normal lung volume loss and consolidation volume increase peaked at 6-months. As time progressed, the regions of consolidation became denser. CASE IV: The increase in high-intensity lung volume remained stable across time-points due to two parallel processes: (1) the presence of a residual mass that shrank across follow-up time-points and (2) progressively increased consolidation volumes. The dashed line highlights the evolution of rotation of the anterior junction line.

Table S.3- P-values for Friedman and post-hoc Wilcoxon tests (with multiple comparison adjustment using Benjamini-Hochberg procedure, 10% false-discovery rate) for CT-based biomarkers across different time-points. Values in bold indicate statistically significant results.

| CT-based biomarker | Friedman test | Pairwise Wilcoxon two-sided signed-rank test | | | | | | |
| --- | --- | --- | --- | --- | --- | --- | --- | --- |
|  | p-value | critical  p-value | uncorrected p-value | | | | | |
|  |  |  | 3-6M | 3-12M | 3-24M | 6-12M | 6-24M | 12-24M |
| $\Delta NV$ | **<0.01** | <0.01 | **<0.01** | **<0.01** | **<0.01** | 0.24 | 0.11 | 0.16 |
| $\mathrm{RV}$ | **0.01** | 0.03 | **0.01** | 0.42 | 0.60 | **<0.01** | **0.03** | 0.41 |
| $\Delta X$ | **<0.01** | 0.01 | **<0.01** | **<0.01** | **<0.01** | **<0.01** | **<0.01** | **0.01** |
| $\Delta Z$ | **0.04** | 0.03 | **0.01** | **0.02** | **0.03** | 0.65 | 0.35 | 0.67 |
| $\Delta h$ | 0.39 | 0.02 | **0.01** | **0.02** | 0.11 | 0.75 | 0.74 | 0.53 |
| $\Delta C$ | **0.04** | 0 | 0.04 | 0.17 | 0.03 | 0.84 | 0.98 | 0.41 |
| $\Delta S$ | 0.32 | 0 | 0.19 | 0.04 | 0.29 | 0.23 | 0.68 | 0.28 |
| $\Delta\alpha$ | **0.08** | <0.01 | 0.07 | 0.04 | **<0.01** | 0.44 | 0.09 | 0.05 |
| $\Delta M$ | **<0.01** | 0.01 | **<0.01** | **<0.01** | **<0.01** | **0.01** | **<0.01** | **<0.01** |
| $\Delta\beta$ | **<0.01** | 0.01 | **<0.01** | **<0.01** | **<0.01** | **0.01** | **<0.01** | **0.01** |
| $\Delta t$ | **<0.01** | 0.06 | **0.06** | **0.04** | **<0.01** | 0.72 | **0.02** | **0.01** |
| $\Delta P$ | **0.01** | 0.01 | **0.01** | 0.79 | 0.21 | **<0.01** | 0.39 | 0.30 |


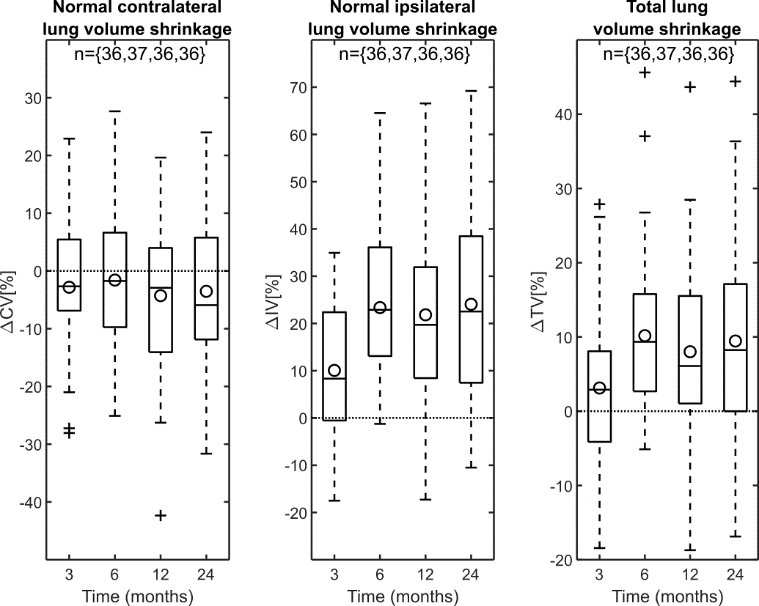


Figure S.3- Relative change (from pre-RT) in normal contralateral lung volume, ipsilateral lung volume, and total lung volume (sum of both normal lung volumes) over serial time-points (3, 6, 12 and 24-months). Positive values indicate loss of volume. Circles indicate average value; horizontal line indicates no change; outliers fall outside the ±2.7std range. Pairs of baseline and follow-up scans acquired with inconsistent inspiration were excluded.

Table S.4- Wilcoxon tests with multiple comparison adjustment (using Benjamini-Hochberg procedure, 10% false-discovery rate) for lung volumes with respect to pre-RT values. Values in bold indicate statistically significant results.

| CT-based lung volumes | Pairwise Wilcoxon two-sided signed-rank test | | | | |
| --- | --- | --- | --- | --- | --- |
|  | critical  p-value | uncorrected p-value | | | |
|  |  | 3M | 6M | 12M | 24M |
| $CV=\mathrm{NV}_{c}$ | 0 | 0.19 | 0.46 | 0.10 | 0.11 |
| $IV=\mathrm{NV}_{i}$ | <0.01 | **<0.01** | **<0.01** | **<0.01** | **<0.01** |
| $TV=\mathrm{NV}_{i}+NV_{c}$ | 0.09 | **0.09** | **<0.01** | **<0.01** | **<0.01** |


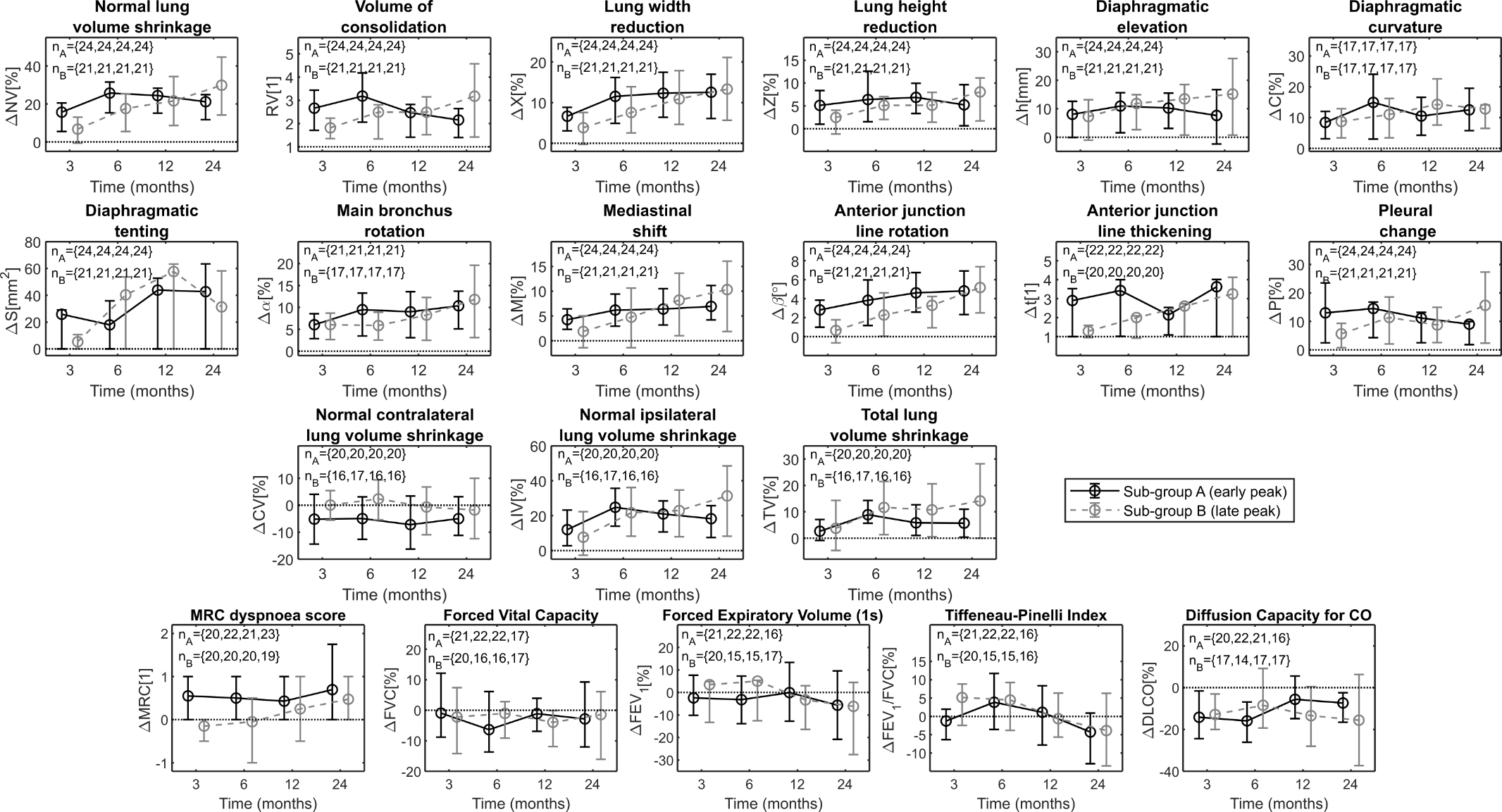


Figure S.4- Average value (±25/75% percentile) for all radiological and PFT data, per sub-group at serial time-points; horizontal line indicates no change.

Table S.5- Wilcoxon tests for baseline characteristics between radiologically defined sub-groups. Values in bold indicate statistically significant results (p≤0.1).

| Factor | Mean (±SD) | Median (Range) | Wilcoxon two-sided rank-sum test p-value |
| --- | --- | --- | --- |
| Age | 65 (±9) | 64 (42 – 83) | 0.75 |
| MRC | 1(±1) | 1 (0 – 3) | **0.01** |
| FVC (%predicted) | 96 (±15) | 95 (70 – 132) | **0.06** |
| FEV_1_ (%predicted) | 77 (±19) | 77 (37 – 117) | 0.10 |
| FEV_1_/FVC (%) | 63 (±12) | 67 (36 – 87) | 0.45 |
| DLCO (%predicted) | 68 (±17) | 67 (42 – 111) | **0.01** |
| Prescribed dose (Gy) | 67.2 (±3.5) | 67.5 (63.0 – 73.0) | 0.42 |
| Mean lung dose (Gy) | 16.5 (±2.6) | 17.0 (9.9 – 22.2) | 0.39 |
| Lung V_5Gy_ (%) | 54 (±10) | 53 (31 – 79) | 0.25 |
| Lung V_10Gy_ (%) | 43 (±9) | 43 (23 – 62) | 0.24 |
| Lung V_20Gy_ (%) | 23 (±6) | 23 (14 – 44) | 0.37 |
| Lung V_40Gy_ (%) | 13 (±3) | 13 (7 – 19) | 0.77 |
| Lung V_60Gy_ (%) | 3 (±1) | 3 (0 – 6) | 0.60 |
| Mean heart dose (Gy) | 9.9 (±7.4) | 9.9 (1.1 –30.8) | 0.43 |
| GTV (cm^3^) | 104 (±76) | 82 (14 – 328) | 0.89 |


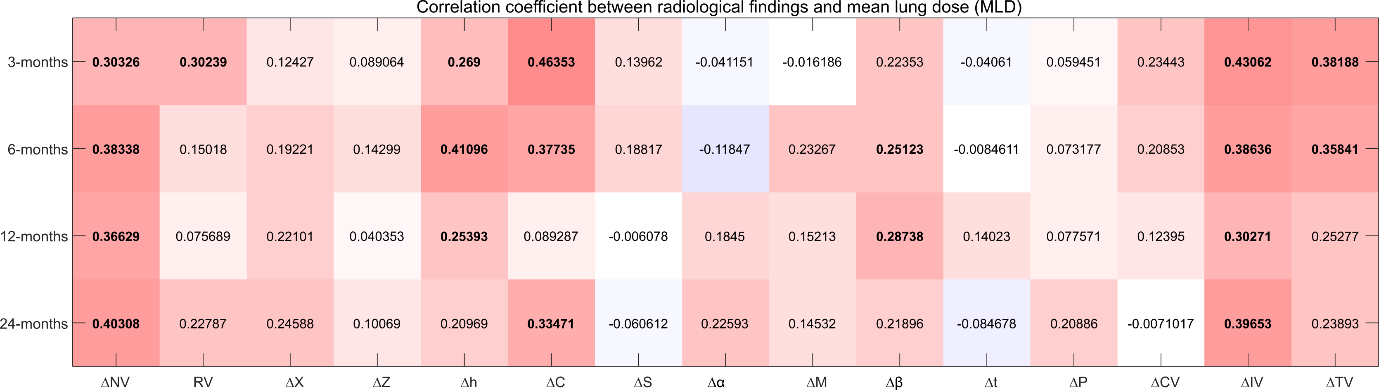


Figure S.5- Pearson’s correlation coefficient between mean lung dose (MLD) and radiological findings over time. Values in bold indicate statistically significant results (p≤0.1). Measures of lung volume shrinkage ($\Delta NV$ and $\Delta IV$) over time correlates consistently and the strongest with global RT dosimetry.


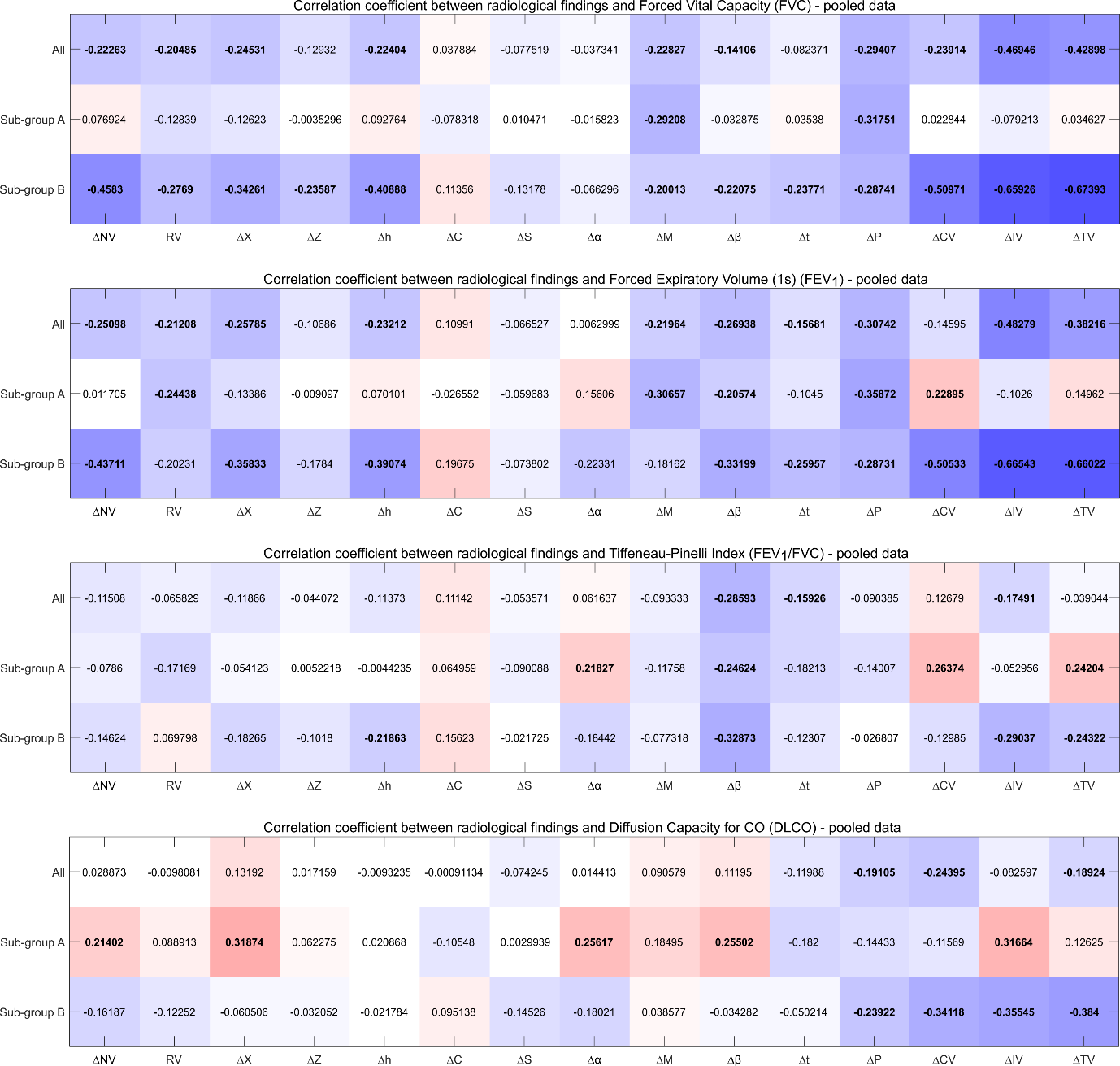


Figure S.6- Pearson’s correlation coefficient between pulmonary function tests (PFTs) and radiological findings (data pooled over all timepoints) for the whole patient group and divided into sub-groups. Values in bold indicate statistically significant results (p≤0.1). Stronger correlations are found for FVC and FEV_1_ with radiological change (in particular with measures of volume shrinkage). Note that relative volume changes from pre-RT values correlate more strongly than relative changes to the contralateral lung. $\Delta$FEV_1_/FVC had poorer correlation with volume changes and correlated best with mediastinal rotation. Similarly, DLCO correlated poorly with radiological findings. In general, there is a stronger correlation between radiological findings and PFTs for sub-group B (late peak).
